# Gestational diabetes and spousal health: the Finnish gestational diabetes study

**DOI:** 10.1093/eurpub/ckag057

**Published:** 2026-04-07

**Authors:** Tea Taskila, Marja Vääräsmäki, Sanna Mustaniemi, Sofia Leppänen, Hannele Laivuori, Eero Kajantie, Elina Keikkala, The FinnGeDi Study Group

**Affiliations:** Research Unit of Clinical Medicine, Department of Obstetrics and Gynecology, Medical Research Center, Oulu University Hospital, University of Oulu, Oulu, Finland; Welfare, Epidemiology and Monitoring Unit, Department of Public Health, Finnish Institute for Health and Welfare, Helsinki and Oulu, Finland; Research Unit of Clinical Medicine, Department of Obstetrics and Gynecology, Medical Research Center, Oulu University Hospital, University of Oulu, Oulu, Finland; Welfare, Epidemiology and Monitoring Unit, Department of Public Health, Finnish Institute for Health and Welfare, Helsinki and Oulu, Finland; Research Unit of Clinical Medicine, Department of Obstetrics and Gynecology, Medical Research Center, Oulu University Hospital, University of Oulu, Oulu, Finland; Welfare, Epidemiology and Monitoring Unit, Department of Public Health, Finnish Institute for Health and Welfare, Helsinki and Oulu, Finland; Research Unit of Clinical Medicine, Department of Obstetrics and Gynecology, Medical Research Center, Oulu University Hospital, University of Oulu, Oulu, Finland; Welfare, Epidemiology and Monitoring Unit, Department of Public Health, Finnish Institute for Health and Welfare, Helsinki and Oulu, Finland; Faculty of Medicine and Health Technology, Center for Child, Adolescent, and Maternal Health Research, Tampere University, Tampere, Finland; Department of Obstetrics and Gynecology, Tampere University Hospital, Wellbeing Services County of Pirkanmaa, Tampere, Finland; Medical and Clinical Genetics, University of Helsinki and Helsinki University Hospital, Helsinki, Finland; Helsinki Institute of Life Science, Institute for Molecular Medicine Finland, University of Helsinki, Helsinki, Finland; Research Unit of Clinical Medicine, Department of Obstetrics and Gynecology, Medical Research Center, Oulu University Hospital, University of Oulu, Oulu, Finland; Welfare, Epidemiology and Monitoring Unit, Department of Public Health, Finnish Institute for Health and Welfare, Helsinki and Oulu, Finland; Children’s Hospital, University of Helsinki and Helsinki University Hospital, Helsinki, Finland; Department of Clinical and Molecular Medicine, Norwegian University of Science and Technology, Trondheim, Norway; Research Unit of Clinical Medicine, Department of Obstetrics and Gynecology, Medical Research Center, Oulu University Hospital, University of Oulu, Oulu, Finland; Welfare, Epidemiology and Monitoring Unit, Department of Public Health, Finnish Institute for Health and Welfare, Helsinki and Oulu, Finland

## Abstract

The spouses of women with gestational diabetes mellitus (GDM) seem to be at risk of developing type 2 diabetes and cardiovascular diseases (CVDs). We comparatively analysed the risk factors, lifestyle, socioeconomic factors, and health of the spouses of women with (cases, *n = *599) and without (controls, *n = *586) GDM. This cross-sectional study utilized data from the Finnish Gestational Diabetes study. Data of the spouses were collected using a structured questionnaire: socioeconomic factors, smoking, alcohol consumption, health, own perinatal health, and family history of diabetes and CVDs. Age-adjusted odds ratios (aORs) were analysed using multivariate logistic regression. The mean ages of the cases and the controls were 33.5 years and 31.2 years, respectively [mean difference: 2.4 years, 95% confidence interval (CI): 1.68–3.02]. The mean body mass index of the cases (26.9 kg/m^2^) was 0.78 kg/m^2^ (95% CI: 0.34–1.21) higher than that of the controls. Fewer cases attained the highest educational level (13.5% vs. 16.9%, aOR 0.64, 95% CI: 0.46–0.90). The cases reported more often alcohol consumption (85.4% vs. 78.7%, aOR 1.59, 95% CI: 1.16–2.17); chronic disease, impairment, or disability (17.4% vs. 12.6%, aOR 1.43, 95% CI: 1.03–2.00); or mental disorder (8.0% vs. 5.1%, aOR 1.63, 95% CI: 1.01–2.64). The cases reported more risk factors for adverse health outcomes, more chronic diseases and mental disorders than the controls. Therefore, lifestyle counselling should also be provided to the spouses of women with GDM.

## Introduction

Gestational diabetes mellitus (GDM) is a form of diabetes that is diagnosed for the first time during pregnancy. It affects about 14% of pregnancies globally [1] and about 20% in Finland [Finnish Institute for Health and Welfare (THL) 2024, unpublished data]. GDM has impacts on the health of both the mother and the child. Women diagnosed with GDM using the International Association of the Diabetes and Pregnancy Study (IADPSG) criteria have a 6.4-fold higher risk of developing type 2 diabetes (T2DM) later in life than women without GDM [2]. They have also been found to develop metabolic syndrome (MetS) and cardiovascular disease (CVD) more often [3]. Compared to children born from euglycemic pregnancies, children antenatally exposed to GDM are more likely to experience obesity, metabolic disturbances, neurodevelopmental disorders, and allergies in childhood and adolescence [4–7].

Just as women with GDM have long-term health risks after their pregnancies, it has been reported that their spouses also have higher risks of developing CVD and metabolic morbidity than those of women without GDM [8]. A cross-sectional study conducted in India found that spouses of women with GDM or diabetes in pregnancy had cardiometabolic risk factors more often than spouses of women without GDM, including overweight [body mass index (BMI) ≥25.0 kg/m^2^ 60.3% vs. 48.7%, and MetS 48.3% vs. 30.0%] [9]. In two Canadian longitudinal retrospective studies based on the same population-based cohort with a mean follow-up time of 13 years, spouses of women with GDM had a 1.2-fold risk of T2DM compared to spouses of women without GDM [10, 11] and a 1.2-fold risk of CVD or all-cause mortality [11]. Furthermore, the findings of an Iranian prospective population-based study with a median follow-up time of 14.1 years suggested that spouses of women with GDM have a 36% higher risk of developing CVD compared to spouses of women without GDM [12]. In a more recent Canadian population-based cohort study, spouses’ risk of diabetes, hypertension, and CVD increased with the number of pregnancy complications (GDM, gestational hypertension, and preeclampsia) [13]. The effects that lifestyle and socioeconomic factors have on spousal morbidity are not fully understood. Hence, we conducted a cross-sectional study to determine whether there are differences in socioeconomic factors, smoking, alcohol use, and the health of spouses of women with and without GDM. We also comparatively analysed the spouses’ perinatal history and family history of T2DM and CVD.

## Methods

### Selection and description of participants

This cross-sectional study is part of the Finnish Gestational Diabetes (FinnGeDi) study. The data were collected between 2009 and 2012 [14], after the implementation of the new Finnish national comprehensive screening guidelines for GDM (Finnish Current Care Guidelines of GDM) in 2008 [15]. Before that, GDM screening was based on the presence of risk factors.

At the time of our study in Finland, GDM screening was offered to all pregnant women except those with a very low risk of GDM: primiparous women under 25 years old with a BMI <25 kg/m^2^ and without a family history of T2DM, and multiparous women under 40 years old with a BMI <25 kg/m^2^ and without previous GDM and a macrosomic newborn (birth weight >4500 g). Screening involved conducting a 2-hour 75-g oral glucose tolerance test (OGTT) after overnight fasting at 24–28 weeks of gestation. For high-risk groups [prior GDM, BMI ≥35.0 kg/m^2^, glucosuria, polycystic ovary syndrome, or T2DM in family history (i.e. parents, grandparents, siblings, or children)], the OGTT was performed at 12–16 weeks of gestation. When this early pregnancy OGTT was normal, the test was repeated at 24–28 weeks of gestation. A diagnosis of GDM was made when at least one test value met or exceeded the cut-off value (fasting level ≥5.3 mmol/L, 1-hour level ≥10.0 mmol/L, or 2-hour level ≥8.6 mmol/L) [15]. Between 2009 and 2012, 51.4% of pregnant women were screened for GDM [14].

The FinnGeDi case–control cohort includes 1146 women diagnosed with GDM and 1066 consecutive women without GDM who gave birth in one of the seven delivery units included in the study [14]. The couples were recruited and signed an informed consent in the delivery unit when the women came in to give birth. All women with GDM were asked to participate in the study, and the consecutive woman without GDM who came in was recruited as a control. Exclusion criteria were maternal pre-pregnancy diabetes and multifetal pregnancy. Data were also collected on the newborns. Both parents were asked to complete a background questionnaire at the time of recruitment. All spouses who completed the questionnaire were included in the present study (1185/2212; 53.6%), with no additional exclusion criteria. The participants of this study were divided into two groups: (i) the GDM group (cases), which consisted of the spouses of women with GDM (599/1146; 52.3%), and (ii) the non-GDM group (controls), which consisted of the spouses of women without GDM (586/1066; 55.0%). The participants’ flow chart is shown in Fig. S1.

This study was conducted in accordance with the Helsinki Declaration and was approved by the Ethics Committee of the Northern Ostrobothnia Hospital District in 2008.

### Data and measurements

The spouses’ data were collected from the questionnaire. The women’s data were obtained from the questionnaire, medical records, and the Finnish Medical Birth Register (MBR). MBR is a national database that includes detailed information on pregnancy, delivery, perinatal health, and the newborn’s health until the age of 7 days. The delivery hospital completes a structured form for the MBR of all live births and stillbirths of infants weighing ≥500 g or having a gestational age of ≥22 weeks.

Each participant’s age was calculated from their reported date of birth and the recruitment date. Data on the following socioeconomic factors at the time of recruitment were collected: educational level, occupational status, marital status, and children from previous relationships (‘yes’ or ‘no’; if yes, the number of children). Educational level was assessed using four categories: basic (comprehensive school), secondary (general upper secondary school, further education college, or college-level institute), lower-level tertiary (post-secondary non-tertiary education or university of applied sciences), and upper-level tertiary (university). In the logistic regression analysis, the other categories were compared to the ‘secondary’ category. Occupational status was assessed using six categories: employment/entrepreneurship, househusband, student, unemployed, pensioner, and other (including military service, sick leave, and rehabilitation). The other categories were compared to the ‘employment/entrepreneurship’ category in the logistic regression analysis. Marital status was defined using five categories: married, cohabitating, divorced, living alone, and living with parents. For the statistical analyses, marital status was dichotomized (married or cohabitating = ‘yes’, others = ‘no’).

Two lifestyle factors were analysed: smoking and alcohol use. The participants were asked to share whether they smoked before and/or during the pregnancy, with the available answers being at least 20 cigarettes daily, 10–19 cigarettes daily, up to nine cigarettes daily, once or more per week but not daily, less than once a week, former smoker, and never smoked. The data were combined and dichotomized to smoking before/during the pregnancy and no smoking (former smoker and never smoked). Alcohol use was assessed using data collected from two questions: (i) How often do you consume alcohol? and (ii) How often do you binge drink? The answer choices for both questions were daily, twice a week, once a week, twice a month, about once a month, once every second month, three or four times a year, once a year or more seldom, and I do not consume alcohol. The answers were grouped into four categories: non-user, about once every couple of months or less frequently, once or twice a month, and once a week or more frequently. In the logistic regression analysis, the other categories were compared to the ‘non-user’ category. In addition to analyse spouses′ alcohol consumption, we further combined and dichotomized the data on alcohol consumption into two categories: (does not consume alcohol = ‘no’, other = ‘yes’).

Each participant’s BMI (kg/m^2^) was calculated from their self-reported height and weight. The following questionnaire item was used to assess the participants’ health: Do you have any chronic disease, impairment, or disability that affects your everyday life? (‘yes’ or ‘no’). A more detailed question was included to collect information on specific diseases, namely T1DM, T2DM, hypertension, dyslipidaemia, coronary artery disease, stroke, and mental disorders (depression, panic disorder, schizophrenia, other psychosis, or other mental disorders), with ‘yes’/’no’ answer options. Mental disorders were analysed as a group. An open-ended question was also included to collect information on medications used (name, dose, date started, and date of last use). For further analysis, we generated a combination variable for chronic diseases, including CVD, dyslipidaemia, and mental disorders, using the self-reported diagnosis and medication data (if the diagnosis or medication = ‘yes’, the combination variable = ‘yes’). In the medications for CVD, we included angiotensin-converting enzyme (ACE) inhibitors, angiotensin 2 receptor blockers (ARBs), diuretics, calcium channel blockers, beta blockers, and acetylsalicylic acid (ASA). Statins were included as dyslipidaemia medications. The medications for mental disorders included selective serotonin reuptake inhibitors (SSRIs), selective serotonin and norepinephrine inhibitors (SNRIs), monoamine oxidase inhibitors (MAOIs), atypical antidepressants, and second-generation antipsychotics.

Data on each participant’s perinatal history were collected, including their gestational age at birth, birth weight, and mother’s preeclampsia and GDM status during the index pregnancy. The participants were also asked to provide some details of their family history, particularly information on their parents’ conditions and cause of death, specified as T1DM, T2DM, and CVD (myocardial infarction, stroke, or hypertension).

For missing participant analysis, we compared the spouses who completed the questionnaire to those who did not complete the questionnaire, based on the characteristics of the women. The women’s characteristics were collected from their medical records or questionnaires, and the data were assessed using the same questions and categorizations as for the spouses detailed above.

### Statistical analysis

Statistical analyses were conducted using IBM SPSS version 29.0. To examine the differences between the groups, continuous variables with normal distribution were analysed with an independent samples *t*-test, and those without normal distribution were analysed using the Mann–Whitney *U* test. Categorical variables were analysed using the χ^2^ test and Fisher’s exact test when the sample size was small (>20% cells with an expected count <5). A two-tailed *P* values <.05 was considered significant. Categorical variables were analysed using logistic regression analyses and reported as odds ratios (ORs) with 95% confidence intervals (CI). Pearson’s correlation was used to analyse the correlations between couples’ characteristics.

## Results

### Age and socioeconomic factors

When we compared the mean ages of the GDM and non-GDM groups, we found that the spouses in the GDM group were 2.4 years (95% CI: 1.68–3.02) older than the spouses in the non-GDM group (33.5 years vs. 31.2 years, *P < .*001). Therefore, age-adjusted ORs (aORs) are reported in the analyses.

Fewer spouses in the GDM group had attained the highest educational level than in the non-GDM group (upper-level tertiary 13.5% vs. 16.9%, aOR 0.64, 95% CI: 0.46–0.90); however, there was no difference in occupational or cohabitating/marital status between the groups. The spouses in the GDM group reported having children from previous relationships more frequently and having more children from such relationships than the spouses in the non-GDM group; however, the former was related to their older age (aOR 1.35, 95% CI: 0.89–2.07 and aOR 1.19, 95% CI: 0.93–1.53). The characteristics of the participants are presented and compared in Table 1.

**Table 1.** Comparison of characteristics of the participants

	GDM group (*n = *599)	Non-GDM group (*n = *586)	OR/mean difference (95% CI)^a^	aOR (95% CI)
Age, years; mean (SD)	33.5 (6.1)	31.2 (5.6)	−2.35 (−3.02 to −1.68)	**−**
Missing, *n* (%)	3 (0.5)	5 (0.9)		
BMI, kg/m²; mean (SD)	26.9 (3.9)	26.2 (3.7)	−0.78 (−1.21 to −0.34)	–
Missing, *n* (%)	6 (1.0)	6 (1.0)		
Educational level				
Basic, *n* (%)	31 (5.2)	35 (6.0)	0.790 (0.477 to 1.31)	0.839 (0.498 to 1.41)
Secondary, *n* (%)	381 (63.6)	340 (58.0)	Reference	Reference
Lower-level tertiary, *n* (%)	105 (17.5)	112 (19.1)	0.837 (0.617 to 1.13)	0.808 (0.593 to 1.10)
Upper-level tertiary, *n* (%)	81 (13.5)	99 (16.9)	0.730 (0.526 to 1.01)	0.639 (0.456 to 0.895)
Missing, *n* (%)	1 (0.2)	0 (0)		
Occupational status				
Employment/entrepreneurship, *n* (%)	534 (89.4)	514 (87.7)	Reference	Reference
Househusband, *n* (%)	5 (0.8)	3 (0.5)	1.60 (0.381 to 6.75)	1.37 (0.315 to 6.01)
Student, *n* (%)	24 (4.0)	38 (6.5)	0.608 (0.360 to 1.03)	0.802 (0.464 to 1.39)
Unemployed, *n* (%)	26 (4.4)	24 (4.1)	1.04 (0.591 to 1.84)	1.27 (0.705 to 2.29)
Pensioner, *n* (%)	6 (1.0)	2 (0.3)	2.89 (0.580 to 14.4)	0.361 (0.064 to 2.03)
Other, *n* (%)	2 (0.3)	5 (0.9)	0.385 (0.074 to 1.99)	1.07 (1.048 to 1.09)
Missing, *n* (%)	2 (0.3)	0 (0)		
Cohabitating/married, *n* (%)	595 (99.3)	585 (99.8)	0.254 (0.28 to 2.28)	0.771 (0.517 to 1.15)
Missing, *n* (%)	0	0		
Children from previous relationships, *n* (%)	71 (12.3)	39 (7.0)	1.87 (1.24 to 2.81)	1.35 (0.877 to 2.07)
Missing, *n* (%)	24 (4.0)	30 (5.1)		
Number of children from previous relationships			1.43 (1.12 to 1.82)	1.19 (0.93 to 1.53)
0	529 (88.3)	546 (93.2)		
1	36 (6.0)	25 (4.3)		
2	24 (4.0)	10 (1.7)		
3 or more	10 (1.7)	5 (0.9)		
Missing, *n* (%)	0	0		

Abbreviations: aOR, age-adjusted odds ratio; BMI, body mass index; CI, confidence interval; GDM, gestational diabetes; OR, odds ratio; SD, standard deviation.

a Mean difference with 95% CI for continuous parameters.

### Lifestyle factors

There were no differences in the smoking habits of the groups. The rate of alcohol consumption was higher in the GDM group (85.4%) than in the non-GDM group (78.7%) (aOR 1.59, 95% CI: 1.16–2.17). There were also more weekly consumers in the GDM group compared to the non-GDM group: 49.7% of the participants in the GDM group reported consuming alcohol once a week or more, whereas 43.5% of the participants in the non-GDM group reported the same frequency of consume (aOR 1.62, 95% CI: 1.17–2.26). In addition, the members of the GDM group reported weekly binge drinking more often than those in the non-GDM group (once a week or more often: 7.7% vs. 3.4%, aOR 3.10, 95% CI: 1.70–5.63) (Table 2).

**Table 2.** Comparison of the lifestyle factors of the participants

	GDM group (*n = *599)	Non-GDM group (*n = *586)	OR (95% CI)	aOR (95% CI)
Smoking				
Before pregnancy, *n* (%)	257 (43.3)	247 (42.4)	1.04 (0.823–1.31)	1.21 (0.948–1.54)
Missing, *n* (%)	5 (0.8)	3 (0.5)		
During pregnancy, *n* (%)	249 (42.2)	233 (39.9)	1.10 (0.872–1.39)	1.26 (0.991–1.61)
Missing, *n* (%)	9 (1.5)	2 (0.3)		
Use of alcohol			1.61 (1.19–2.18)	1.59 (1.16–2.17)
Yes, *n* (%)	512 (85.4)	461 (78.7)		
No, *n* (%)	85 (14.2)	123 (21.0)		
Missing, *n* (%)	2 (0.33)	2 (0.34)		
Alcohol consumption				
Once a week or more often, *n* (%)	298 (49.7)	254 (43.5)	1.70 (1.23–2.35)	1.62 (1.17–2.26)
Once or twice a month, *n* (%)	140 (23.4)	159 (27.0)	1.27 (0.891–1.82)	1.35 (0.936–1.95)
Once every second month or more seldom, *n* (%)	74 (12.4)	48 (8.2)	2.23 (1.41–3.52)	2.11 (1.32–3.37)
Non-user, *n* (%)	85 (14.2)	123 (21.1)	Reference	Reference
Missing, *n* (%)	2 (0.3)	2 (0.4)		
Binge drinking				
Once a week or more often, *n* (%)	46 (7.7)	20 (3.4)	3.35 (1.86–6.03)	3.10 (1.70–5.63)
Once or twice a month, *n* (%)	179 (29.9)	196 (33.4)	1.33 (0.955–1.85)	1.37 (0.973–1.92)
Once every second month or more seldom, *n* (%)	279 (46.6)	231 (39.4)	1.760 (1.284–2.41)	1.75 (1.27–2.41)
Non-user, *n* (%)	94 (15.7)	137 (23.4)	Reference	Reference
Missing, *n* (%)	1 (0.2)	2 (0.4)		

Abbreviations: aOR, age-adjusted odds ratio; CI, confidence interval; GDM, gestational diabetes; OR, odds ratio.

### Spousal health and morbidity

**Figure 1.**
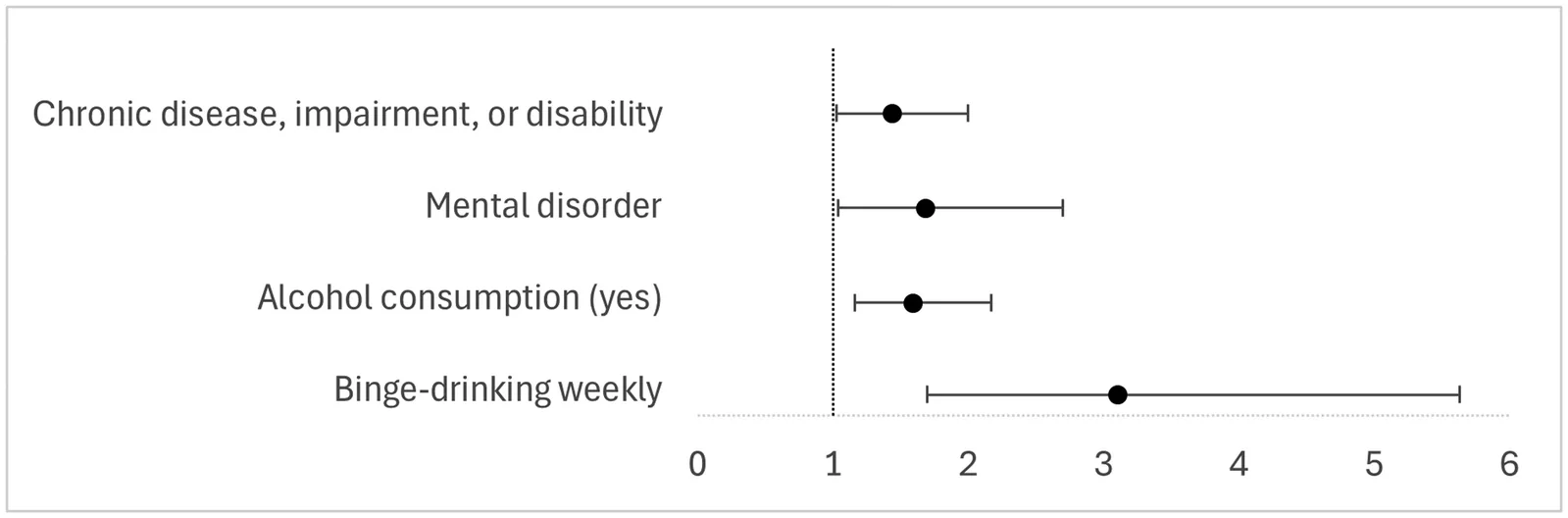
Age-adjusted odd ratios for the association of spousal health outcomes with maternal gestational diabetes status using logistic regression analysis. The variables include self-reported data about chronic disease, impairment, or disability that affects everyday life, mental disorder, including diagnosis and medication, alcohol consumption frequency, which is dichotomized to ‘yes’ and ‘no’, and weekly binge-drinking. All variables are compared between the spouses of the GDM group and the non-GDM group.

The spouses of the women with GDM had higher BMI values than those of the women without GDM (26.9 kg/m^2^ vs. 26.2 kg/m^2^, mean difference: 0.78 kg/m^2^, 95% CI: 0.34–1.21). They also reported having more chronic diseases, impairments, or disabilities (17.4% vs. 12.6%, aOR 1.43, 95% CI: 1.03–2.00). There were no differences between the groups in terms of the prevalence of CVD, T2DM, and dyslipidaemia. The spouses in the GDM group reported experiencing mental disorders more often than those in the non-GDM group (8.2% vs. 5.1%, aOR 1.68, 95% CI: 1.04–2.70) (Table 3 and Fig. 1).

**Table 3.** Comparison of the participants’ morbidity and medication data

	GDM group (*n = *599)	Non-GDM group (*n = *586)	OR (95% CI)	aOR (95% CI)
Any chronic disease, impairment, or disability, *n* (%)	104 (17.4)	74 (12.6)	1.47 (1.06–2.03)	1.43 (1.03–2.00)
T1DM, *n* (%)	7 (1.2)	4 (0.7)	1.149 (0.917–1.440)	1.119 (0.887–1.410)
T2DM, *n* (%)	6 (1.0)	0 (0.0)	1.19 (0.948–1.50)	1.16 (0.918–1.47)
CVD^a^, *n* (%)	44 (7.3)	21 (3.6)	2.13 (1.25–3.63)	1.52 (0.874–2.63)
Hypertension, *n* (%)	27 (4.5)	11 (1.9)		
CAD, *n* (%)	1 (0.2)	0		
Stroke, *n* (%)	1 (0.2)	0		
Medication for CVD, *n* (%)	37 (6.2)	21 (3.6)		
Dyslipidaemia^b^, *n* (%)	53 (8.8)	44 (7.5)	1.20 (0.788–1.81)	0.930 (0.603–1.44)
Dyslipidaemia diagnosis, *n* (%)	52 (8.7)	42 (7.2)		
Medication for dyslipidaemia, *n* (%)	15 (2.5)	7 (1.2)		
Mental disorder^c^, *n* (%)	49 (8.2)	30 (5.1)	1.65 (1.03–2.64)	1.68 (1.04–2.70)
Mental disorder diagnosis, *n* (%)	48 (8.0)	30 (5.1)		
Mental disorder, medication, *n* (%)	19 (3.2)	13 (2.2)		

Abbreviations: aOR, age-adjusted odds ratio; BMI, body mass index; CAD, coronary artery disease; CI, confidence interval; CVD, cardiovascular disease; GDM, gestational diabetes; OR, odds ratio; T1DM, type 1 diabetes; T2DM, type 2 diabetes; SD, standard deviation.

a Includes self-reported diagnosis of hypertension, coronary artery disease, and stroke and any medication for these conditions.

b Includes self-reported diagnosis and medication for dyslipidaemia.

c Includes self-reported diagnosis and medication for mental disorder (excluding sedatives).

### Perinatal and family medical histories

No differences were observed between the groups in terms of spousal prenatal exposure to preeclampsia or GDM. The spousal gestational age at birth and birthweight values also did not differ between the groups. The prevalence of parental diabetes or CVD did not differ between the study groups (Table S1).

### Women’s characteristics

Women in the GDM group were older, had higher BMI, lower educational level, and were less often primiparous than controls. No difference was found in occupational and cohabiting/marital status or smoking status before/during pregnancy between the groups. In the GDM group, 20.2% had pharmacological treatment for GDM. The mode of delivery was more often caesarean section, and the newborns were more likely to be large for gestational age in the GDM group than in the control group (Table S2).

### Correlation with maternal parameters

Correlations were observed between the couple’s age, BMI educational level, and smoking during pregnancy. Spouse’s CVD correlated with the woman’s chronic hypertension. Correlations between spouses’ morbidity and women’s hypertensive pregnancy complications were not observed (Table S3).

### Missing participant data analysis

The women whose spouses completed the questionnaire were younger, had a slightly higher educational level, smoked, and cohabitated more often than the women whose spouses did not complete the questionnaire. The women’s pre-pregnancy BMI, occupational status, and GDM status did not differ between the groups (Table S4).

## Discussion

The results of this cross-sectional study revealed two novel findings. First, the spouses of women with GDM not only had more risk factors for adverse health outcomes but also more chronic diseases, impairments, disabilities, or mental disorders than the spouses of women without GDM. Second, it was evident that these participants had developed these conditions at a relatively young age.

Regarding the occurrence of risk factors for adverse health outcomes, our finding that the BMI values of the spouses of women with GDM were higher than those of the spouses of women without GDM aligns with those of previous studies [9, 12]. However, our results show that the participants in the GDM group also developed more morbidities (chronic diseases and mental disorders) despite still being relatively young (early thirties). Due to the low number of cases included in this study, the type of mental disorder was not assessed. In a previous study, Pace *et al.* [16] found no concordance between a woman’s GDM status and the incidence of spousal depression.

In the current study, 85.4% of the participants in the GDM group and 78.7% of the participants in the non-GDM group reported that they consumed alcohol, and 7.7% of the participants in the GDM group and 3.4% of the participants in the non-GDM group reported that they binge drank once a week or more often. Spousal alcohol consumption was not assessed in previous studies conducted in Canada, Iran, and India [9–13]. In general, excessive alcohol consumption is related to several adverse health outcomes. Therefore, we investigated alcohol consumption in our cohort. Alcohol consumption may vary between countries and cultures. In a population-based study in Finland, 94.5% of men aged 20–54 years consumed alcohol: 61% consumed it weekly and 19% binge drank weekly in 2008 [17]. These proportions are higher than those found in our study. These differences may have been due to our participants having active family lives and thus using alcohol less often than the broader group of men represented in the national register data, which included men of different ages and in different life situations. In addition to other findings reflecting an unhealthier lifestyle, our observation on increased alcohol consumption suggests that spouses in the GDM group might have a more complex socioeconomical burden and cumulation of risk factors of adverse health outcomes than controls.

When we examined the occurrence of selected socioeconomic factors, we found that the spouses of women with GDM were slightly older and slightly less educated but that their occupational status did not differ from the spouses of women without GDM. Our results are comparable to those reported in the literature [12].

As expected, we found correlations between the couples for the following factors: age, BMI, educational level, and smoking status during pregnancy. Previous studies have indicated that lifestyle factors and patterns, such as maintaining a healthy body weight and eating habits, spread within social networks and tend to converge over the course of time or a relationship [18, 19]. This may partly be due to non-random mating: People tend to partner with those who share the same social environment and have a similar lifestyle, including in terms of eating habits and level of physical activity [20]. We also found that the spouses of women with chronic hypertension had CVD more often, which may indicate the severity of vascular morbidity in these couples. Unlike Mussa *et al.* [13], we found no correlation between spouses’ morbidity and women’s gestational hypertension and preeclampsia in our study.

Our findings suggest that GDM is associated with spousal health. The spouses of women with GDM were observed to have a slightly increased risk of adverse health outcomes compared to the spouses of women without GDM. However, the spouses’ family history of diabetes and CVD and perinatal characteristics did not differ between the study groups. These important findings should inform clinical work and future research. In addition, they highlight the need to extend counselling services to fathers during and after pregnancies with GDM, which are mainly provided to mothers with GDM. Brazeau *et al.* [21] highlighted that spouses of women with a recent GDM history can be recruited to T2DM prevention programmes to help them adopt healthy changes to their lifestyle patterns. A previous study also showed that spousal co-participation in a T2DM prevention programme after a pregnancy with GDM could enhance the participation of women and improve outcomes [22]. Thus, family context is important in pregnancy and postpartum counselling, and family-wide counselling might improve both the outcomes for women with GDM and their spouses’ risk factors for T2DM and CVD, as well as help prevent or at least postpone their onset. Health services could utilize this information by extending preventive healthcare screening and counselling to the spouses of women with GDM at the time of GDM diagnosis.

### Strengths and limitations

The strengths of the present study are that the FinnGeDi study data are well documented and that the size of our cohort was reasonable. The women in the GDM group represented a typical Finnish GDM phenotype. Also, more than half of the spouses involved in the FinnGeDi study completed the questionnaire and were included in the present study. However, our study was based on self-reported data collected using a questionnaire, which can be considered a limitation. Some selection bias might have existed in our study. According to our missing participant data analysis, spouses who completed the questionnaire were slightly younger than those who did not. Furthermore, the women partnered with the men who participated in the study and completed the questionnaire had a higher educational level and smoked more frequently (before or during pregnancy) than the women partnered with the men who did not complete the questionnaire, implying that there may be differences in the socioeconomic factors between the participants and non-participants. Hence, the spouses included in this study may not fully represent the original FinnGeDi study population. Nonetheless, according to the missing participant data analysis, there was no difference in the women’s pre-pregnancy BMI, educational level, occupational status, marital status, or GDM status between the spouses who did and did not participate in this study.

## Conclusions

In this study, the spouses of women with GDM had more risk factors for adverse health outcomes and more often reported diseases, impairments, and disabilities that affected their everyday lives, and mental disorders than the spouses of women without GDM. These differences were already evident at a relatively young age. Therefore, the spouses of women with GDM should be provided counselling at the time of the GDM diagnosis and during the post-pregnancy follow-up period, alongside the mothers. In addition to enhancing spousal health outcomes, this action may also improve the participation rate of women with prior GDM in follow-up programmes and, hence, the health of the whole family.

## Supplementary Material

ckag057_Supplementary_Data

## Data Availability

The data underlying this article cannot be shared publicly due to the privacy of individuals who participated in the study. Key Points The spouses of women with GDM have higher BMI values and more risk factors for adverse health outcomes than those of women without GDM. The spouses of women with GDM more often report having diseases, impairments, and disabilities that affect their everyday lives, and mental disorder than those of women without GDM. The family context should be emphasised during counselling of women with GDM and counselling extended to spouses of women with GDM.
