# Supplementary material for: Gestational diabetes and spousal health: the Finnish gestational diabetes study
Source: Eur J Public Health. 2026 Apr 7;36(2):ckag057. doi: 10.1093/eurpub/ckag057 (PMC13061638; doi:10.1093/eurpub/ckag057)
Supplement: ckag057_Supplementary_Data [file ckag057_supplementary_data.zip › ejph-2025-11-om-0968-File007.docx]

**Supplementary Table S1.** Participants’ perinatal characteristics and family history of diabetes and CVD.

|  | GDM group  (n = 599) | Non-GDM group  (n = 586) | OR (95% CI) | aOR (95% CI) |
| --- | --- | --- | --- | --- |
| Gestational age at birth, weeks, mean (SD)  Missing, n (%) | 39.59 (1.78)  209 (34.9) | 39.71 (1.83)  192 (32.8) | 0.113 (−0.140 - 0.367) | - |
| Birth weight, g, mean (SD)  Missing, n (%) | 3605 (571)  120 (20.0) | 3611 (587)  116 (19.8) | 5.60 (−68.2 – 79.4) | - |
| Pregnancy complications during mother’s pregnancy, n (%)  Preeclampsia, n (%)  Missing or not known, n (%)  GDM, n (%)  Missing or not known, n (%) | 11 (11.9)  138 (23)  12 (2.1)  142 (23.7) | 8 (1.4)  133 (22.7)  4 (0.7)  127 (21.7) | 0.951 (0.727 – 1.24)  1.02 (0.777 – 1.34) | 0.951 (0.723 – 1.25)  0.987 (0.747 – 1.31) |
| Parental diabetes, n (%)  Missing, n (%) | 109 (18.2)  66 (11.0) | 107 (18.3)  97 (16.6) | 0.996 (0.741 – 1.34) | 0.857 (0.632 – 1.16) |
| Parental CVD, n (%)  Missing, n (%) | 287 (47.9)  236 (39.4) | 244 (41.6)  296 (50.5) | 1.289 (1.03 - 1.62) | 1.092 (0.86 - 1.39) |

aOR: age-adjusted odds ratio

CI: confidence interval

CVD: cardiovascular disease

GDM: gestational diabetes

OR: odds ratio
