# Supplementary material for: Gestational diabetes and spousal health: the Finnish gestational diabetes study
Source: Eur J Public Health. 2026 Apr 7;36(2):ckag057. doi: 10.1093/eurpub/ckag057 (PMC13061638; doi:10.1093/eurpub/ckag057)
Supplement: ckag057_Supplementary_Data [file ckag057_supplementary_data.zip › ejph-2025-11-om-0968-File008.docx]

| **Supplementary Table S2**. Comparison of the characteristics of the women with or without GDM among the spouses who completed the questionnaire (n = 1185). |  |  |  |  |  |
| --- | --- | --- | --- | --- | --- |
| Women’s characteristics |  | GDM group | non-GDM group | p-value^a^ |  |
|  |  | n = 599 | n = 586 |  |  |
| Age, years; mean (SD)^b^ |  | 31.7 (5.3) | 29.1 (4.8) | <0.001 |  |
| Prepregnancy BMI, kg/m²; mean (SD)^c^ |  | 28.1 (5.9) | 23.8 (4.1) | <0.001 |  |
| Educational level^de^ |  |  |  | 0.048 |  |
|  | Basic, n (%) | 46 (7.7) | 43 (2.5) |  |  |
|  | Secondary, n (%) | 288 (48.1) | 278 (47.4) |  |  |
|  | Lower-level tertiary, n (%) | 155 (25.9) | 153 (26.1) |  |  |
|  | Upper-level tertiary, n (%) | 110 (18.4) | 130 (22.2) |  |  |
|  | Missing, n (%) | 0 | 0 |  |  |
| Occupational status^e^ |  |  |  | 0.332 |  |
|  | Upper white-collar worker | 106 (20.3) | 118 (23.1) |  |  |
|  | Lower white-collar worker | 239 (45.9) | 208 (40.8) |  |  |
|  | Blue-collar worker | 65 (12.5) | 61 (12.0) |  |  |
|  | Other | 111 (21.3) | 123 (24.1) |  |  |
| Smoking before pregnancy^d^ |  |  |  | 0.694 |  |
|  | no | 408 (68.3) | 406 (69.4) |  |  |
|  | yes | 189 (31.7) | 30.6) |  |  |
| Smoking during pregnancy^e^ |  |  |  | 0.205 |  |
|  | no | 496 (82.8) | 501 (85.5) |  |  |
|  | yes | 103 (17.2) | 85 (14.5) |  |  |
| Domestic partnership^e^ |  |  |  | 0.709 |  |
|  | Yes | 568 (94.8) | 557 (95.1) |  |  |
|  | No | 24 (4.0) | 25 (4.3) |  |  |
|  | Unknown | 7 (1.2) | 4 (0.7) |  |  |
| Marital status^e^ |  |  |  | 0.116 |  |
|  | married | 390 (65.1) | 370 (63.1) |  |  |
|  | unmarried | 203 (33.9) | 215 (36.7) |  |  |
|  | widowed | 0 | 0 |  |  |
|  | divorced | 6 (1.0) | 1 (0.2) |  |  |
|  | registered | 0 | 0 |  |  |
| Primipara^e^ |  |  |  | <0.001 |  |
|  | no | 360 (60.1) | 290 (49.5) |  |  |
|  | yes | 239 (39.9) | 296 (50.5) |  |  |
| Chronic hypertension^cf^ |  |  |  | <0.001 |  |
|  | no | 502 (83.8) | 558 (95.2) |  |  |
|  | yes | 97 (16.2) | 28 (4.8) |  |  |
| Gestational hypertension^cg^ |  |  |  | 0.002 |  |
|  | no | 467 (78.0) | 498 (85.0) |  |  |
|  | yes | 132 (22.0) | 88 (15.0) |  |  |
| Preeclampsia^ch^ |  |  |  | <0.001 |  |
|  | no | 559 (93.3) | 576 (98.3) |  |  |
|  | yes | 40 (6.7) | 10 (1.7) |  |  |
| Pharmacologically treated GDM^c^ |  |  |  |  |  |
|  | no | 121 (20.2) | 586 (100) |  |  |
|  | yes | 478 (79.8) | 0 |  |  |
| Mode of delivery^e^ |  |  |  | <0.001 |  |
|  | Vaginal delivery | 476 (79.5) | 534 (91.1) |  |  |
|  | Caesarean section | 123 (20.5) | 52 (8.9) |  |  |
| Large for gestational age^ei^ | |  |  | <0.001 |  |
|  | no | 504 (84.1) | 546 (93.2) |  |  |
|  | yes | 95 (15.9) | 40 (6.8) |  |  |
|  |  |  |  |  |  |
|  |  |  |  |  |  |
|  |  |  |  |  |  |
|  |  |  |  |  |  |
| ^a^ Student’s t-test used for continuous variables, and Pearson’s χ2 test used for categorical parameters | | | | | |
| ^b^ Age at the time of delivery |  |  |  |  |  |
| ^c^ data from medical records |  |  |  |  |  |
| ^d^ data from the questionnaire |  |  |  |  |  |
| ^e^ data from the Finnish Medical Birth Record (MBR) | |  |  |  |  |
| ^f^ Systolic blood pressure ≥ 140 mmHg and/or diastolic blood pressure ≥ 90 mmHg detected before 20 weeks of gestation. | | | | | |
| ^g^ Blood pressure ≥ 140/90 mmHg, no proteinuria. | |  |  |  |  |
| ^h^ Blood pressure ≥ 140/90 mmHg and proteinuria (≥ 0.3 g /24h or two ≥ 1+ readings on a dipstick). | | | |  |  |
| ^i^ Birthweight >90 percentile |  |  |  |  |  |
|  |  |  |  |  |  |
