# Supplementary material for: Gestational diabetes and spousal health: the Finnish gestational diabetes study
Source: Eur J Public Health. 2026 Apr 7;36(2):ckag057. doi: 10.1093/eurpub/ckag057 (PMC13061638; doi:10.1093/eurpub/ckag057)
Supplement: ckag057_Supplementary_Data [file ckag057_supplementary_data.zip › ejph-2025-11-om-0968-File009.docx]

**Supplementary Table S3.** Correlations of the medical conditions between the couple.

|  |  |  | Women's variables | |  |  |  |  |
| --- | --- | --- | --- | --- | --- | --- | --- | --- |
| P-values for the correlations |  |  |  |  |  |  |  |  |
| Spouses' variables | Age | BMI | Educational level | Smoking during pregnancy | CH | GH | PE |  |
| Ageª | <0.0001 |  |  |  |  |  |  |  |
| BMI |  | <0.0001 |  |  |  |  |  |  |
| Educational level |  |  | <0.0001 |  |  |  |  |  |
| Smoking during pregnancy |  |  |  | <0.0001 |  |  |  |  |
| Any chronic disease, impairment, or disability |  |  |  |  | 0.693 | 0.346 | 1.000 |  |
| CVDᵇ |  |  |  |  | 0.027 | 0.816 | 0.360 |  |
| Dyslipidaemiaᶜ |  |  |  |  | 0.225 | 0.421 | 0.792 |  |
| Mental disorder ͩ |  |  |  |  | 0.340 | 0.459 | 0.139 |  |
|  |  |  |  |  |  |  |  |  |
| P-value based on Pearson Chi-square for categorical variables and Pearson correlation test for continuous variables | | | | | | | |  |
|  |  |  |  |  |  |  |  |  |
| ª age at the time of recruitment for the spouses and age at the time of delivery for the women | | | | | |  |  |  |
| BMI: body mass index, kg/m² | |  |  |  |  |  |  |  |
| CH: chronic hypertension, systolic pressure constantly ≥ 140 mmHg and/or diastolic blood pressure ≥90 mmHg before 20+0 weeks of gestation, 1=yes, 0=no | | | | | | | | |
| GH: gestational hypertension, systolic blood pressure ≥ 140 mmHg and/or ≥ 90 mmHg ONLY after 20 weeks of gestation, 1=yes, 0=no | | | | | | | | |
| PE: preeclampsia, systolic blood pressure ≥ 140 and/or diastolic blood pressure ≥ 90 and proteinuria ≥ 300 mg/day or two ≥ 1+ readings on a dipstick , 1=yes, 0=no | | | | | | | | |
|  |  |  |  |  |  |  |  |  |
|  |  |  |  |  |  |  |  |  |
| ᵇCVD = Includes self-reported diagnosis of hypertension, coronary artery disease, and stroke and, any medication for these conditions | | | | | | | | |
| ᶜ Includes self-reported diagnosis and medication for dyslipidaemia | | | |  |  |  |  |  |
| ͩ Includes self-reported diagnosis and medication for mental disorder (excluding sedatives) | | | | | |  |  |  |
