# Supplementary material for: Gestational diabetes and spousal health: the Finnish gestational diabetes study
Source: Eur J Public Health. 2026 Apr 7;36(2):ckag057. doi: 10.1093/eurpub/ckag057 (PMC13061638; doi:10.1093/eurpub/ckag057)
Supplement: ckag057_Supplementary_Data [file ckag057_supplementary_data.zip › ejph-2025-11-om-0968-File010.docx]

Supplementary Table S4. Missing participants’ data. Comparison of women´s (with or without GDM) characteristics between the spouses who completed (yes) and did not complete the questionnaire (no).

|  |  |  | Spouses’ Questionnaire | | | |  |
| --- | --- | --- | --- | --- | --- | --- | --- |
|  |  |  | No |  | Yes |  | p value |
|  |  |  | N | % | N | % |  |
|  |  |  | 1027 | 46.4 | 1185 | 53.6 |  |
| Women´s occupational status |  |  |  |  |  |  |  |
|  | Upper white-collar worker | | 175 | 23.7 | 224 | 21.7 |  |
|  | Lower white-collar worker | | 312 | 42.2 | 447 | 43.4 |  |
|  | Blue-collar worker | | 107 | 14.5 | 126 | 12.2 |  |
|  | Other |  | 145 | 19.6 | 234 | 22.7 | 0.219 |
| Women´s smoking* | | |  |  |  |  |  |
|  | no |  | 752 | 73.6 | 813 | 68.6 |  |
|  | yes |  | 270 | 26.4 | 372 | 31.4 | 0.010 |
| Women´s educational attainment |  |  |  |  |  |  |  |
|  | basic | | 39 | 5.0 | 71 | 6.0 |  |
|  | secondary | | 346 | 44.4 | 566 | 47.8 |  |
|  | lower-level tertiary | | 193 | 24.7 | 308 | 26.0 |  |
|  | Upper-level tertary | | 202 | 25.9 | 240 | 20.3 | 0.030 |
| Women´s GDM | | |  |  |  |  |  |
|  | no |  | 480 | 46.7 | 586 | 49.5 |  |
|  | yes |  | 547 | 53.3 | 599 | 50.5 | 0.203 |
| Pharmacologically treated GDM |  |  |  |  |  |  |  |
|  | no |  | 927 | 90.3 | 1064 | 89.8 |  |
|  | yes |  | 100 | 9.7 | 121 | 10.2 | 0.711 |
| Domestic partnership | | |  |  |  |  |  |
|  | yes |  | 850 | 82.8 | 1125 | 95.0 |  |
|  | no |  | 62 | 6.0 | 49 | 4.1 |  |
|  | unknown | | 114 | 11.1 | 10 | 0.8 | 0.000 |
| Marital status | | |  |  |  |  |  |
|  | married | | 608 | 59.2 | 760 | 64.1 |  |
|  | unmarried | | 407 | 39.6 | 418 | 35.3 |  |
|  | widowed | | 1 | 0.1 | 0 | 0.0 |  |
|  | divorced | | 9 | 0.9 | 7 | 0.6 |  |
|  | registered | | 2 | 0.2 | 0 | 0.0 | 0.060 |
| p value based on Pearson Chi-square | | | |  |  |  |  |
| *before or during the pregnancy | | |  |  |  |  |  |

|  | Questionnaire | | | | | | Difference | | |
| --- | --- | --- | --- | --- | --- | --- | --- | --- | --- |
|  | No |  |  | Yes |  |  | Mean | 95% CI |  |
|  | N | Mean | SD | N | Mean | SD |  | Lower | Upper |
| Women´s age, years | 1027 | 31.4 | 5.62 | 1185 | 30.4 | 5.21 | 0.98 | 0.53 | 1.43 |
| Women´s pre-pregnancy BMI, kg/m² | 1027 | 26.0 | 5.89 | 1184 | 26.0 | 5.56 | 0.03 | -0.45 | 0.51 |
| Spouses´ age, years | 740 | 33.3 | 6.15 | 1177 | 32.4 | 5.98 | 0.97 | 0.41 | 1.53 |
|  |  |  |  |  |  |  |  |  |  |

BMI: Body mass index

CI: Confidence interval

GDM: gestational diabetes

p value based on Student´s T-test

SD: Standard deviation
