# Supplementary figures and images for: Gestational diabetes and spousal health: the Finnish gestational diabetes study
Source: Eur J Public Health. 2026 Apr 7;36(2):ckag057. doi: 10.1093/eurpub/ckag057 (PMC13061638; doi:10.1093/eurpub/ckag057)

Figure S1. Participants’ flow chart.


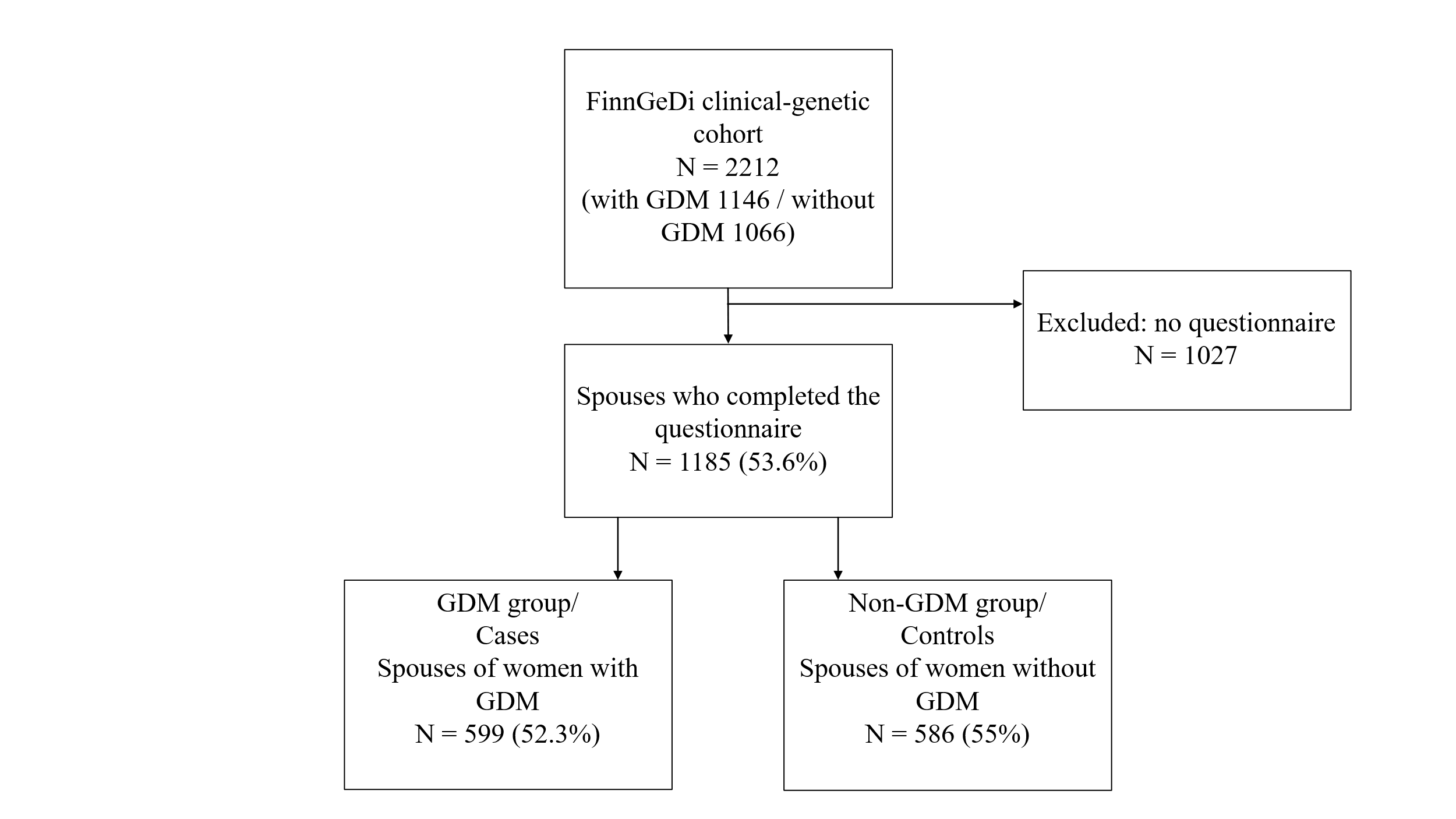

Supplement: ckag057_Supplementary_Data [file ckag057_supplementary_data.zip › ejph-2025-11-om-0968-File006.docx]
